# Editorial: Post-translational Modifications and Compartmentalized Protein Quality Control in Cardiac Muscle and Disease

**DOI:** 10.3389/fphys.2021.745887

**Published:** 2021-08-30

**Authors:** Mark J. Ranek, Aldrin V. Gomes, Huabo Su

**Affiliations:** ^1^Department of Medicine, Division of Cardiology, The Johns Hopkins Medical Institutions, Baltimore, MD, United States; ^2^Neurobiology, Physiology, and Behavior Department, University of California, Davis, Davis, CA, United States; ^3^Department of Pharmacology and Toxicology, Vascular Biology Center, Augusta University, Augusta, GA, United States

**Keywords:** post-translational modification, protein quality control, heart failure, mitochondria, endoplasmic reticulum

The proteome regulates the development, functioning, and adaptation of cells and organisms over time. Maintenance of a functional proteome is critical for the health of all cell types, especially to those with limited regeneration capacity such as cardiomyocytes. Cardiac development and maturation is accompanied by a drastic change in the composition of cardiac proteome due to increasing protein synthesis and a switch of fetal-to-adult protein expression (Rowton et al., 2021). Similarly, cardiac stress induces a dramatic alteration in the cardiac proteome that in turn has a significant impact on cardiac remodeling. Not surprisingly, derangement of the cardiac proteome is linked to various cardiomyopathy and heart failure (Lau et al., 2018). Thus, a better understanding of how cardiac structure and function is regulated at the protein level is crucial in delineating the molecular mechanisms underlying cardiac disease.

Cells maintain protein homeostasis (proteostasis) through an elegant protein quality control (PQC) system that involves targeted proteolysis and diverse protein post-translational modifications (PTMs). Proteolysis via the ubiquitin proteasome system (UPS) and autophagy offers the first line of PQC by removing unneeded normal proteins and misfolded/damaged proteins (Wang et al., 2008), which if not promptly removed will perturb other proteins, organelles, and cellular processes, causing cell malfunction and death. By directly controlling protein half-lives, targeted proteolysis is considered a coarse and bulk regulation of proteostasis. In contrast, a growing list over 300 different types of PTMs can serve as a fine-tuned PQC mechanism by regulating the stability, activity, subcellular localization and binding affinity to proteins/DNA of diverse protein substrates, thus having pleiotropic effects on various cellular processes. Partly due to these PTMs the complexity of the proteome is much larger than the genome. Targeted proteolysis and PTMs can occur at various subcellular compartments such as cytosol, mitochondria, and endoplasmic reticulum (ER) to preserve the normal function of these organelles. Inadequate targeted proteolysis and aberrant PTMs are known to contribute to impaired cardiac development and function, ultimately leading to failure (Wang et al., 2013; Willis and Patterson, 2013). Thus, improving cellular PQC has potential as a new therapeutic strategy for cardiac protection. Recent advances have identified new PTMs and compartmentalized proteolysis as novel avenues to protect the myocardium during development and disease. These exciting studies opened up new area of research. The present Research Topic, *Post-Translational Modifications and Compartmentalized Protein Quality Control in Cardiac Muscle and Disease*, highlights recent advances in our understanding of how various PTMs and compartmentalized proteolysis impact protein homeostasis and PQC pathways, along with discussing the promising roles of these discoveries in cardiac pathophysiology and therapeutic potential.

Cardiac development requires an intricately regulated process of protein expression and degradation to facilitate the genesis of the left ventricle/atrium from the first heart field and the right ventricle/atrium from the second heart field. We are beginning to understand the proteins involved in this complex process and even more recently, the PTMs influencing the proteins of this process. Chen et al. reviews the role of the Hippo pathway, including Yes-associated protein 1 (YAP1) and PDZ-binding motif (TAZ), during cardiac development, disease, and regeneration. While the Hippo pathway is conserved and has been shown to regulate organ size and the response of the heart to stress, only recently have PTMs such as phosphorylation, O-GlcNacylation, methylation, and ubiquitination been identified to regulate this pathway during development and disease (Chen et al.).

Cardiac pressure overload induces cardiomyocyte hypertrophy and proteotoxic stress, both of which can be attenuated through the activation of autophagy. Wu et al. demonstrate one of the mechanisms that autophagy protects the myocardium is by controlling nuclear factor erythroid factor 2-related factor (Nrf2). This exciting observation adds clarity to a field that was mystified as to whether Nrf2 was protective or detrimental. Multiples studies demonstrated the cardioprotection afforded by Nrf2; however other studies revealed Nrf2 is associated with the progression of cardiomyopathies. Here the authors propose that when autophagy is intact and can be stimulated, that activation of Nrf2 is protective; whereas when autophagy is inhibited Nrf2 is detrimental by driving the expression of angiotensinogen, which in turn promotes angiotensin II production (Wu et al.).

Compartmentalized regulation of organelle specific quality control mechanisms is a burgeoning area of research and has demonstrated therapeutic potential to attenuate cardiac disease. Mitochondria are tasked with producing enough energy for the heart to function, a challenge that is greater during disease. Indeed, mitochondrial dysfunction is a common hallmark of cardiovascular disease (Quiles and Gustafsson; Fan et al.). Cardiomyocytes possess quality control mechanisms to maintain mitochondrial quality control as reviewed by Quiles and Gustafsson and Fan et al. Mitochondrial health is a balance between mitochondrial biogenesis, fission, fusion, and mitophagy as the cardiomyocyte attempts to limit reactive oxygen species production, while meeting the energy demands of the heart. Mitochondrial quality control is achieved via the UPS, mitochondrial unfolded protein response, and mitophagy (Quiles and Gustafsson). PTMs regulate not only the stability of the proteins of the mitochondria but also the regulatory systems to increase mitochondrial health (Fan et al.). These manuscripts highlight targets for enhancing mitochondrial quality control mechanisms and the potential therapeutic benefit to treat cardiac disease. Endoplasmic reticulum (ER) proteostasis includes the unfolded protein response, which is initiated upon the accumulation of misfolded protein in ER lumen. Glembotski et al. reviews the role of one of the ER membrane proteins, ATF6, whose activation protects the myocardium by enhancing cardiomyocyte PQC. Seminal discoveries attributed a protective response of ATF6 activation to both inside and outside of the ER along with identifying novel small molecule activators of ATF6 to enhance proteostasis (Glembotski et al.).

Myofilament contraction is central to generate the force needed for cardiomyocytes to fulfill its mechanical function. The requirement of the heart to eject blood into the circulation places the sarcomeres of the cardiac cells under constant stress. Li et al. demonstrate Slit2, a secreted glycoprotein, protects the myocardium during ischemia reperfusion injury by inhibiting the inflammatory response to maintain myofilament contraction. The underlying mechanism for this protection was Slit2 expression inhibited the nuclear translocation of NFκB p65 along with decreasing IL-1β and IL-18 release (Li et al.).

The last few decades have seen many advances in our understanding of the mechanisms regulating cardiac PQC. Lewno et al. review the involvement of Cullin-RING ligases (CRLs) during cardiomyocyte necroptosis, focusing on the role of cullin neddylation (activates CRLs) and deneddylation by the COP9 signalosome. Downregulation of cullin deneddylation is associated with increased cardiac proteotoxicity and necroptosis during stress (Su et al., 2011). Wiley et al. explores the role of arginylation, mediated by arginyltransferase1 (ATE1), in the heart. They uncovered ATE1 interacts with select intracellular nodules important for cardiac physiology, namely translation/transcription regulation, biosynthesis, cell morphology, response to oxidative stress, and mitochondrial function (Wiley et al.). A major PTM, phosphorylation, is known to regulate many proteins associated with maintaining cardiomyocyte proteostasis. Mishra et al. review important cardiac proteins known to be phosphorylated, how this PTM regulates the proteins, the role of these modified proteins in cardiac patho-physiology, and potential therapeutic targets for cardiac disease.

This Research Topic illustrates exciting and significant advances the field of regulating cardiac PQC has made, and provides novel therapeutic targets that can be leveraged in the clinic for cardiac disease. Our understanding of these molecular mechanisms underlying cardiac proteostasis networks are continuously expanding and becoming better defined. Discovering key signaling nodes allows the field to identify and pursue new targets for therapeutic intervention against cardiac disease.

## Author Contributions

MR drafted and edited the editorial. AG reviewed and revised the editorial. HS planned, edited and finalized the editorial, and response to editors' report. All authors contributed to the article and approved the submitted version.

## Conflict of Interest

The authors declare that the research was conducted in the absence of any commercial or financial relationships that could be construed as a potential conflict of interest.

## Publisher's Note

All claims expressed in this article are solely those of the authors and do not necessarily represent those of their affiliated organizations, or those of the publisher, the editors and the reviewers. Any product that may be evaluated in this article, or claim that may be made by its manufacturer, is not guaranteed or endorsed by the publisher.
